# Digital Framing in End-of-Life Communication: Constructing “Good Death” Support in the Discourse of Hospice Care Institutions in the UK and Hong Kong

**DOI:** 10.3390/healthcare14091187

**Published:** 2026-04-28

**Authors:** Yau Ni Wan, Gail Forey, Winnie Zeng

**Affiliations:** 1 Department of English Language and Literature, Hong Kong Shue Yan University, 10 Wai Tsui Crescent, Braemar Hill, North Point, Hong Kong; ynwan@hksyu.edu; 2 Department of Education, University of Bath, Bath BA2 7AY, UK; g.forey@bath.ac.uk; 3 Department of Language Science and Technology, The Hong Kong Polytechnic University, 11 Yuk Choi Road, Hung Hom, Kowloon, Hong Kong

**Keywords:** digital health communication, hospice care, end-of-life, cross-cultural comparison, sentiment and interpersonal engagement, mixed-methods approach

## Abstract

**Background:** Hospice refers to specialised end-of-life care that supports patients and families, making it an important area for studying how language shapes experiences and expectations of care. This study compares hospice discourse on websites in Hong Kong and the United Kingdom, analysing how NLP-based sentiment and interpersonal features, such as personal pronouns and conjunctions, shape logical relations, structure information, and express emotion in patient narratives. **Methods:** Using a mixed approach that integrates sentiment analysis with Systemic Functional Linguistics (SFL), and taxonomy of conjunctions in particular, this study draws on a 52,086-word corpus from 40 hospice websites (20 from each region). The corpus analytical tool AntConc was used to identify co-occurrence, interpret log-likelihood, and perform concordance analysis. **Results:** The findings reveal significant differences in the digital delivery of hospice care across regions. According to our data, UK websites tend to express a wider range of personal emotions and frequently use concessive conjunctions when discussing sensitive palliative care topics. In contrast, Hong Kong websites tend to use more additive and causal conjunctions, projecting a stronger focus on institutional care. For example, Hong Kong texts tend to use formal, service-oriented connections such as “we + offer”, reflecting a more informational communicative style. However, both regions frequently use personal pronouns such as “you” and “we” to convey positive sentiment and demonstrate empathy towards patients and their caregivers. **Conclusion:** These patterns appear to be used strategically by hospice providers to build trust, signal alignment, and strengthen relationships tailored to each region. Lastly, this study makes an original contribution by combining computational and functional linguistic approaches to develop a systematic method for examining culturally shaped digital communication in end-of-life contexts, thereby enriching the field of healthcare discourse analysis.

## 1. Introduction

Hospice discourse is situated at the intersection of factual medical information and affective emotional support, driven by social and cultural values surrounding caregiving and care-receiving as well as the broader meanings attached to death [1,2]. With the rapid development of digital platforms used by the healthcare sector, hospice service centre websites have become an important channel for sharing palliative information, empathy, and institutional values with patients and their caregivers [3,4,5]. In these spaces, where emotional support intersects with practical guidance, language plays a significant role in shaping perceptions of hospice care, building trust, guiding decision-making and educating individuals and families through the complex stages of end-of-life care [6]. In cross-cultural contexts, it is important to understand how service providers use language to construct the appropriate emotional style and interpersonal meaning [7]. This task is complex because attitudes towards end-of-life matters, emotional expression, familial responsibility, and support differ significantly between individuals, communities, and cultures [8], influencing how hospice institutions ultimately frame and shape their digital communication.

This study focuses on hospice websites from two regions with distinct cultural traditions and linguistic practices regarding palliative care: Hong Kong (HK) and the United Kingdom (UK). The aim is to investigate how language features, such as quantitative sentiment polarity, qualitative conjunctions, and personal pronouns, shape the interpersonal dimension of digital hospice discourse. This is a hybrid, mixed-methods approach that combines natural language processing (NLP)-based sentiment analysis with the Systemic Functional Linguistics (SFL) framework. This enables us to discover computational and linguistic patterns of domain frequency and emotion polarity, to further investigate the statistical significance of the functional roles of specific linguistic features, and also illustrate how service providers signal empathy and emotional shifts in ways consistent with culturally embedded end-of-life values in a 52,086-word corpus.

The present study discusses three interrelated linguistic resources: sentiment, conjunctions, and personal pronouns. The first research question examines how sentiment subjectivity and polarity are distributed across hospice websites in HK and the UK. Then, we identify how dominant attitudinal patterns derived from cultural differences affect meaning potentials in end-of-life healthcare communication.

**RQ1:** *How do sentiment polarity and subjectivity differ in official hospice websites in HK and the UK, and what do these differences reveal about culturally shaped approaches to communicating end-of-life care?*

Then, building on taxonomy of conjunctions, we examine the structural transitions and textual coherence of concessive (e.g., “however”), additive (e.g., “and”, “also”), and causal (e.g., “because”) conjunctions. This analysis helps us to investigate how language cohesion contributes to the empathetic and persuasive communication style of hospice discourse, and directly answers the second research question:

**RQ2:** *Which conjunctions are dominant in the hospice texts, and how do they relate to sentiment polarity and emotional shifts in hospice discourse?*

Finally, we explore the types and frequency of personal pronouns and their functions in the two different regions. At this stage, we are interested in how service providers in HK and the UK use different patterns of pronouns such as “I”, “we”, “you”, and “they” to establish interpersonal alignment, and how they express empathy when discussing difficult and negative topics such as end-of-life care. Correlating pronoun usage with the preceding sentiment and conjunction analyses enables a holistic interpretation of these linguistic choices, offering insight into cultural attitudes regarding institutional voice and role relationships, which answers our third research question:

**RQ3:** *Which personal pronouns appear frequently in the dataset and are statistically significant, and what role do they play in creating interpersonal meaning in hospice communication?*

Our interdisciplinary study has both academic and practical implications for understanding the construction of culturally sensitive digital health communication in end-of-life care settings across regions, where both practical healthcare information and interpersonal meanings are equally important. This paper is structured as follows: Section 2 reviews the background of digital hospice communication and key studies on sentiment, conjunctions, pronouns, and cross-cultural discourse. Section 3 outlines the methodology, including corpus construction, analytical tools, and coding procedures, and Section 4 presents and discusses the findings in relation to SFL theory. This paper concludes with limitations, implications for hospice practice, and important future research directions.

## 2. Literature Review

Due to the rapid development of digital communication, the World Wide Web has long been a vital platform for receiving and disseminating information with different text and multimedia features to advance users’ accessibility, capture global attention, and encourage public engagement [9,10]. In particular, websites offer rich interactive and adaptive content, which is effective for mass health communication [11,12]. Hospice care plays an essential role in enhancing the quality of life for people with life-limiting illnesses by addressing their physical, emotional, and social needs c.f. [13,14,15]; it focuses on the early identification and alleviation of physical, psychosocial, and spiritual suffering [16,17]. Based on this context, hospice websites provide information and services in regions with ageing populations and evolving healthcare needs. In the Quality of Death Index by the Lien Centre for Palliative Care [18], the UK was ranked first out of eighty-one countries for end-of-life care, while HK was ranked ninth. These rankings reflect the ongoing commitment of both regions to providing high-quality hospice care. Generally, their services include pain and symptom relief, emotional support, and prompt communication between service providers and patients. However, the underlying operating mechanisms of these two regions differ significantly; for example, in the UK, hospice care is supported by the National Health Service (NHS), which integrates hospice provision into primary and community services through national strategies [19,20]. Most hospice centres are operated by charities and are provided free of charge to patients.

Hospice services in HK remain government- and hospital-centric, with limited community-based provision, and fragmented policy coordination [21]. Existing research has primarily examined issues of accessibility, service standards, and cultural attitudes towards end-of-life care within hospice communication [22,23,24]. Recently, however, some studies have analysed the content and usability of local hospice websites in the digital era [25], revealing shortcomings regarding emotional style, cultural adaptation, and user-centred design [26]. In contrast, research in the UK has focused on the needs of marginalised groups, such as homeless individuals and ethnic minorities, as well as on the improvement of overall policy development [27,28,29,30]. Despite the existing literature, very few studies have conducted a cross-national comparison of hospice websites using a big data corpus approach that quantitatively and qualitatively investigates hospice discourse to address the significant and growing need for systemic change. Such comparative work is essential, timely, and necessary for understanding and improving how digital platforms can better support a diverse ageing population in navigating end-of-life care, a universally significant stage for individuals and their families.

Recent studies have employed lexicon-based and machine learning methods, such as sentiment analysis, to investigate emotional patterns in various medical narratives and public health communications [31]; however, the sentiment polarity in digital hospice communication has not yet been fully explored in detail. Sentiment analysis has the advantage of objectively quantifying emotional expressions in a healthcare context, revealing how language can influence attitudes and responses in end-of-life texts [32], which can go on to impact the decision-making process for patients. In addition, the present study draws on SFL to analyse meaning through Halliday’s three metafunctions [33], concentrating on the interpersonal metafunction to explore how linguistic choices construct social roles and relationships. The interpersonal metafunction concerns how language establishes relationships and conveys and negotiates attitudes within a real-world social context. Personal pronouns are particularly important here, as their use significantly impacts empathy and attitudinal connection in digital hospice discourse between service providers and patients.

Moreover, conjunctions play a vital role in managing discourse coherence and signalling emotional shifts between topics and attitudes, thereby contributing to the text’s overall emotional and interpersonal tone [34]. Conjunctions are used to build structured discourse, guiding readers through different texts, content, and emotional transitions [35,36]; they connect ideas and topics and establish the emotional flow within texts. Halliday and Hasan [37] categorise conjunctions as additive, adversative, causal, or temporal, with each type contributes differently to meaning-making [34] (Table 1). This taxonomy offers a valuable framework for understanding how conjunctions facilitate coherence and illuminate emotional expression in hospice communications. In particular, adversative and concessive conjunctions often signal shifts in sentiment or introduce emotional complexity. In the Findings and Discussion Section, we explore how these types of conjunctions function within hospice discourse and interact with sentiment polarity.

The present study explores the use of personal pronouns, such as the inclusive “we”, which fosters a sense of solidarity and alignment [38,39]. In contrast, the use of “you” can either personalise or create distance, depending on the context [40,41]. In hospice discourse, for example, pronouns are frequently used to build empathy and trust. They can also affect perceptions of shared experiences, thereby influencing the emotional impact of hospice communication. Lastly, research indicates that attitudes towards death and dying vary significantly across cultures, shaping how hospice care is communicated [42,43,44]. The present study aims to address gaps in the existing literature by providing a comprehensive analysis of how cultural contexts influence the linguistic features of hospice websites in Hong Kong and the UK. We employ Systemic Functional Linguistics (SFL) as a theoretical framework and use an NLP-based sentiment tool to address the above research niches by investigating how interpersonal meanings are conveyed through language on hospice websites.

## 3. Methodology

To explore digital hospice communication, the study takes a mixed-methods approach, combining computational sentiment analysis with qualitative interpersonal analysis. A corpus of 52,086 words of English-language text was collected from 40 public hospice websites in Hong Kong and the United Kingdom between January and March 2025. The overall corpus consists of 52,086 English words, including the “About Us”, “Our People” and “Our Services” sections of Hong Kong websites. By contrast, the UK corpus is larger and consists of 38,837 words, including contributions from the “About Us”, “Our People” and “Our Services” sections. However, despite the overall size difference between the two regions, both include parallel functional website sections that enable systematic comparison of interpersonal language patterns across institutional contexts. In addition, the AntConc and log-likelihood corpus tools are designed to compare corpora of different sizes; thus, these two datasets are comparable. The websites were selected according to the following criteria: (1) publicly accessible institutional hospice websites without the need for a login or password, (2) availability of English-language content intended for patients, families or caregivers, and (3) provision of regular hospice or palliative care services. Some websites were excluded if they consisted of duplicate content from hospital websites without clear hospice sections, or only provided contact details in the form of brief email or telephone entries. During the pre-processing stage, footers, metadata, navigational menus, policy disclaimers, and other non-discursive features were removed to ensure the analysis focused on meaningful communicative content. The aim was to include major hospice service centers that maintain common communicative practices and organizational models representative of each region. The compiled corpus was therefore designed to reflect the dominant communication patterns of public hospice websites in Hong Kong and the UK.

The corpus consists of public, patient-facing hospice websites operated by government or non-profit institutional service providers in Hong Kong and the United Kingdom. These websites are key channels for disseminating digital information about hospice care philosophies, values, and services to patients, families, and carers. To maintain ethical integrity and focus solely on language discourse in a sensitive end-of-life context, the identities of the websites are not disclosed. The analysis was limited to official websites and excluded user-generated content, independently submitted patient narratives, and conversations on interactive platforms in Frequently Asked Questions sections. Our study examines how hospice institutions construct end-of-life communication in digital public spaces, with no focus on how individual users respond to or engage with these platforms.

Sentiment analysis of the corpus was conducted using TextBlob version 0.19.0, a Python library built on the Natural Language Toolkit (NLTK). TextBlob implements a rule-based, lexicon-driven approach for sentiment analysis rather than a transformer-based model, making it lightweight, transparent, and suitable for small, cross-regional datasets without domain-specific training. The analysis involved quantifying two dimensions:

(1) Sentiment polarity: This dimension was given a continuous score from −1.0 (highly negative) to +1.0 (highly positive), indicating the emotional valence of the text.
(2) Subjectivity: This dimension was given a score from 0.0 (purely objective) to 1.0 (highly subjective), identifying whether the text presents information or personal opinions and feelings.

To validate accuracy on our corpus, two coders manually annotated a random subset of 100 sentences (50 from UK pages, 50 from HK pages) for polarity (negative, neutral, or positive) and subjectivity (objective or subjective). Inter-rater agreement was substantial for both dimensions (polarity: Cohen’s κ = 0.81; subjectivity: Cohen’s κ = 0.86), indicating reliable human coding. TextBlob achieved 87% agreement (Cohen’s κ = 0.77) with consensus human coding for polarity and 86% for subjectivity (Cohen’s κ = 0.72). These values are acceptable for exploratory cross-corpus comparison, though we acknowledge the limitations of a general-purpose lexicon. Nevertheless, we interpret the quantitative results cautiously and complement them with qualitative textual analysis to provide a more nuanced understanding of cross-cultural differences between the Hong Kong and UK corpora.

The sentiment patterns allowed for macro-level quantitative mapping of emotional tone across the corpus. At the micro-level, a comparative statistical analysis was then conducted to identify any significant differences in the distribution of sentiment between the Hong Kong and UK sub-corpora. To qualitatively explore the rhetorical and interpersonal functions of conjunctions and personal pronouns, AntConc was used to perform the analyses. We also conducted log-likelihood tests to statistically measure significant differences in feature frequency between the two sub-corpora. A collocation analysis of the entire corpus was conducted to identify frequent conjunctions and pronouns alongside the findings of the sentiment analysis. The critical values of the log-likelihood test were interpreted using the following established thresholds: LL of 15.13 corresponds to *p* < 0.0001, LL ≥ 10.83 corresponds to *p* < 0.001, LL ≥ 6.63 corresponds to *p* < 0.01, and LL ≥ 3.84 corresponds to *p* < 0.05.

Additionally, concordance analysis was employed to examine the contextual usage of conjunctions and pronouns, revealing their roles in structuring emotional transitions and fostering interpersonal engagement within hospice contexts. We categorised conjunctions using Halliday and Hasan’s model [37] and guided pronoun analysis through Systemic Functional Linguistics, specifically the interpersonal metafunction. To ensure the reliability of the functional annotation of conjunctions and pronouns, two trained coders independently annotated a 10% sample of the corpus. Any discrepancies were resolved through discussion and consensus, after which the annotation guidelines were refined before the full corpus was analysed. All data used in this study were publicly available on institutional hospice websites, and no personal or sensitive information was collected. This study adheres to the ethical standards of digital discourse analysis, including respect for institutional representation and cultural sensitivity when interpreting end-of-life communications.

## 4. Findings and Discussion

This section presents the major findings of the analysis of Hong Kong and UK hospice websites, focusing on how language reflects values and communication strategies in end-of-life care. Section 4.1, Section 4.2 and Section 4.3 draw on sentiment analysis, discourse markers, and personal pronoun usage to explore how emotions, textual cohesion, and interpersonal meanings are constructed in the datasets.

### 4.1. Sentiment Analysis of the Hospice Websites in Hong Kong and the UK

**Figure 1:**
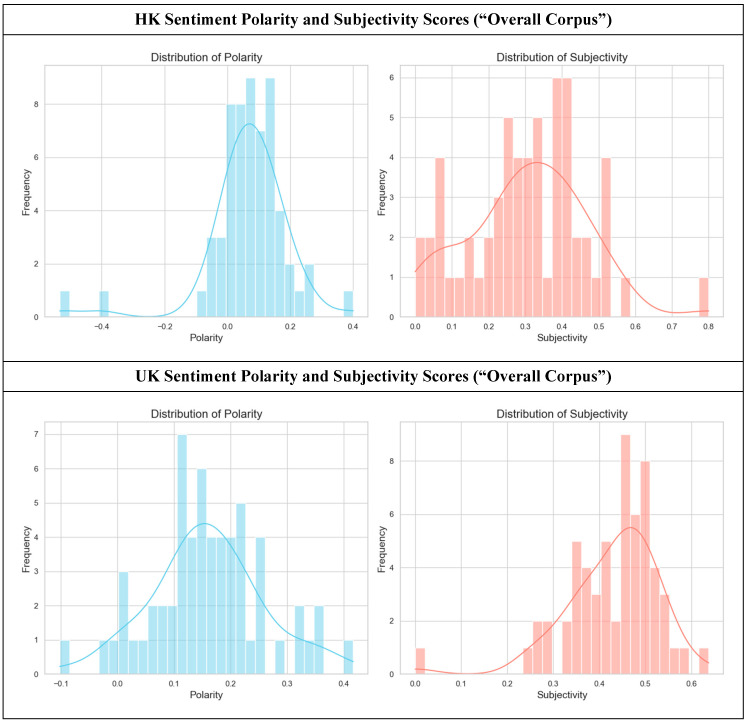
Sentiment polarity and subjectivity scores of hospice websites (HK overall corpus, *n* = 60, vs. UK overall corpus, *n* = 60). A Mann-Whitney U Test indicated that the UK corpus demonstrated significantly higher sentiment polarity (*U* = 940.0, *z* = −4.51, *p* = 0.00001, *r* = 0.41) and subjectivity (*U* = 865.5, *z* = −4.90, *p* = 0.00000, *r* = 0.45) compared to the HK corpus.

An analysis of the sentiment polarity and subjectivity of official hospice websites in HK and the UK reveals different communication strategies. The quantitative data, visualized in the distribution graphs, show a clear divergence in both the emotional tone and the degree of personal expression used to discuss end-of-life care. Figure 1 shows the comparison of the sentiment polarity and subjectivity in HK and the UK.

The sentiment analysis from the HK hospice corpus (top row, *n* = 60) indicates potential communication strategies characterized by emotional restraint and objectivity. The polarity distribution is concentrated around a neutral-to-slightly positive peak (M = 0.07, SD = 0.13, *n* = 60). The sharp, narrow curve signifies low variance, suggesting that the vast majority of the language avoids strong emotional valence, either positive or negative, thus pointing to a preference for a formal, factual, and reassuringly stable tone. For instance, website text might focus on logistical details such as “our facility provides 24-h nursing support” (neutral polarity) or use mildly positive, service-oriented language, such as “we offer a clean and peaceful environment” (low positive polarity). Concurrently, the subjectivity scores for the HK data are spread across the lower-to-mid range (M = 0.31, SD = 0.16, *n* = 60). This suggests a blend of objective statements (e.g., descriptions of medical services, visiting hours) and moderately subjective content that expresses a viewpoint without being overtly personal or emotional. A typical example is “We believe that every patient deserves a dignified journey,” which conveys a belief (subjective) in a formal, institutional voice. The overall impression is one of professional dependability, where trust is built through information and quiet competence rather than overt emotional appeal.

In contrast, the UK hospice corpus (bottom row, *n* = 60) demonstrates a strategy relatively centred on emotive warmth and personal connection. The polarity distribution is visibly shifted towards the positive (M = 0.16, SD = 0.1000, *n* = 60), and the curve is broader, indicating a greater diversity and intensity of positive language. This suggests a communication style that actively uses emotionally resonant words to frame hospice care as a positive, life-affirming experience. For example, UK websites are more likely to feature phrases such as “We help you live life to the fullest” or “Our compassionate team brings comfort and joy to families.” This emphasis on emotion is strongly corroborated by the subjectivity scores. The UK distribution is clearly skewed towards higher subjectivity (M = 0.43, SD = 0.10, *n* = 60), indicating the high prevalence of language that expresses personal feelings, experiences, and opinions. This aligns with a communication strategy that prioritises narrative and personal testimony. One could expect to find patient stories that use highly affective language, such as “I felt so loved and cared for here,” or descriptions that emphasize the personal qualities of the staff, such as “Our amazing nurses are dedicated to making every moment special.”

The Mann-Whitney U Test further revealed that the UK corpus had a significantly higher sentiment polarity than the HK corpus (*U* = 940.0, *z* = −4.51, *p* = 0.00001, *r* = 0.41). The difference in subjectivity was also statistically significant (*U* = 865.5, *z* = −4.90, *p* = 0.00000, *r* = 0.45). The divergence between the two corpora illuminates fundamentally different approaches to communicating about end-of-life care. The Hong Kong strategy of a more restrained positivity and objectivity aligns with cultural tendencies that may prioritise emotional discretion and respect for formality, especially regarding sensitive topics such as death. The goal seems to be to inform and reassure through professionalism and stability. Conversely, in our data, the UK strategy of emotive positivity and high subjectivity reflects a communication style that values emotional transparency and the power of personal narrative. By framing the hospice experience in terms of love, joy, and individual fulfilment, these websites aim to connect and comfort through relatable human emotion. The objective is not just to destigmatize death but to rebrand the end-of-life journey as a meaningful and positive chapter. This approach seeks to build trust by fostering a sense of a warm, caring community.

**Figure 2:**
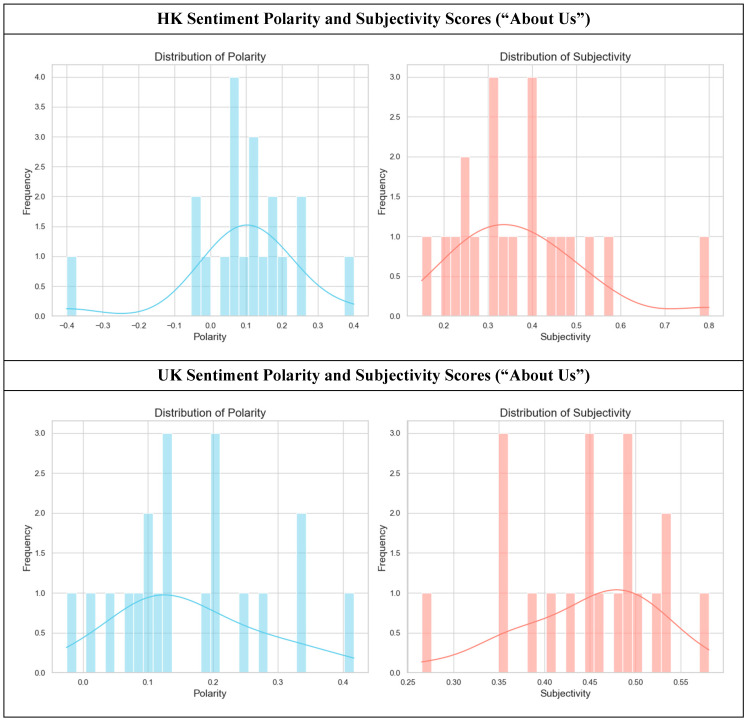
Sentiment polarity and subjectivity scores of hospice websites (HK “About Us”, *n* = 20, vs. UK “About Us” sections, *n* = 20). A Mann-Whitney U Test indicated that UK “About Us” pages had higher polarity (statistically non-significant) compared to the HK pages (*U* = 143.0, *z* = −1.54, *p* = 0.126431, *r* = 0.24). An independent-samples *t*-test indicated that UK “About Us” pages had higher subjectivity (statistically nonsignificant) compared to the HK pages (*t*(38) = −1.99, *p* = 0.05346, Cohen’s d = 0.63).

When concentrating on the specific sections of the hospice websites, the sentiment comparison results reinforce the distinct cultural communication patterns observed in the overall corpora of the two regions. In the “About Us” sections (Figure 2), the language used to construct institutional identity differs between the HK and UK websites.

The HK “About Us” pages (top row, *n* = 20) display a controlled and uniform tone. The polarity distribution is peaked at a slightly positive value (M = 0.093, SD = 0.16), indicating a consistent, formal voice focused on institutional mission and history. The corresponding subjectivity scores in HK are concentrated in the low-to-mid range (M = 0.37, SD = 0.15), suggesting that credibility is likely established through factual statements about the organization’s purpose and services, rather than personal passion or emotional appeal. The UK “About Us” pages (bottom row, *n* = 20) display a more positive tone with higher polarity scores (M = 0.16, SD = 0.11) and higher subjectivity scores (M = 0.45, SD = 0.08), which signifies a potential communication strategy that defines the organization through its values, ethos, and the passionate commitment of its people.

We conducted a Mann-Whitney U Test to compare these distributions, revealing non-significant differences in sentiment polarity (*U* = 143.0, *z* = −1.54, *p* = 0.126431, *r* = 0.24). An independent samples *t*-test was conducted, and the difference in subjectivity is also nonsignificant (*t*(38) = −1.99, *p* = 0.05346, Cohen’s d = 0.63) between the UK and HK “About Us” sections. Despite these null findings, a qualitative inspection of the “About Us” sections suggests a contrast in rhetorical strategy: HK websites tend to foreground institutional authority and factual competence, whereas UK websites construct a narrative of shared values and compassionate dedication to forge a more immediate emotional connection with the reader. 

**Figure 3:**
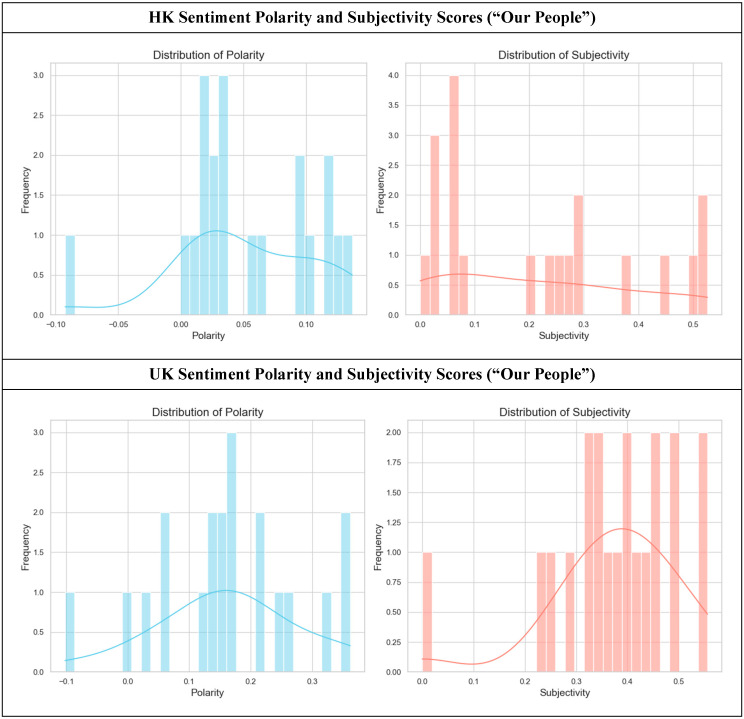
Sentiment polarity and subjectivity scores in hospice websites (HK “Our People”, *n* = 20, vs. UK “Our People” sections, *n* = 20). A Mann-Whitney U test confirmed that the UK texts exhibited significantly higher subjectivity (*U* = 98.5, *z* = −2.75, *p* = 0.006279, *r* = 0.43). A Welch’s *t*-test indicated that the UK texts’ sentiment polarity was significantly higher (*t*(27.24) = −3.77, *p* = 0.00081, Cohen’s *d* = 1.20) compared to the HK texts when describing personnel.

Figure 3 compares the sentiments in the “Our People” sections, in which the portrayal of hospice staff reveals a divergence in communication strategy, moving from professional credentials to personal characteristics.

The HK “Our People” pages (top row, *n* = 20) display a positive value (M = 0.05, SD = 0.06), with personnel using a more positive tone. The subjectivity graph shows a dominant peak at the low end (M = 0.22, SD = 0.18), indicating that staff are primarily defined by their professional roles and qualifications. For instance, descriptions likely focus on titles such as “Dr. Chan, Head of Palliative Care” or “Our team consists of registered nurses,” which are factual and carry a neutral-to-mildly positive polarity. The strategy is to build trust by demonstrating the medical service team’s expertise, professionalism, and competence, and showing that they can provide patients with the best possible service. By contrast, the UK “Our People” pages (bottom row, *n* = 20) use more subjective language to describe their staff (M = 0.37, SD = 0.13), while the polarity is consistently and more intensely positive (M = 0.16, SD = 0.12).

A Mann-Whitney U test confirmed that subjectivity in the UK “Our People” sections was significantly higher than in the HK equivalent (*U =* 98.5, *z =* −2.75, *p =* 0.006279, *r =* 0.43). Simultaneously, the polarity is consistently and more intensely positive, which also represents a statistically significant difference from the HK corpus (Welch’s *t*-test: *t*(27.24) = −3.77, *p* = 0.00081, Cohen’s *d* = 1.20). This suggests that UK websites focus on the personal qualities of their teams, using emotive words such as “compassionate”, “caring”, and “dedicated”. The narrative shifts from what the staff are (regarding their titles) to who they are (regarding their character). This strategy builds trust by fostering an emotional connection and portraying the team as a warm, supportive family.

**Figure 4:**
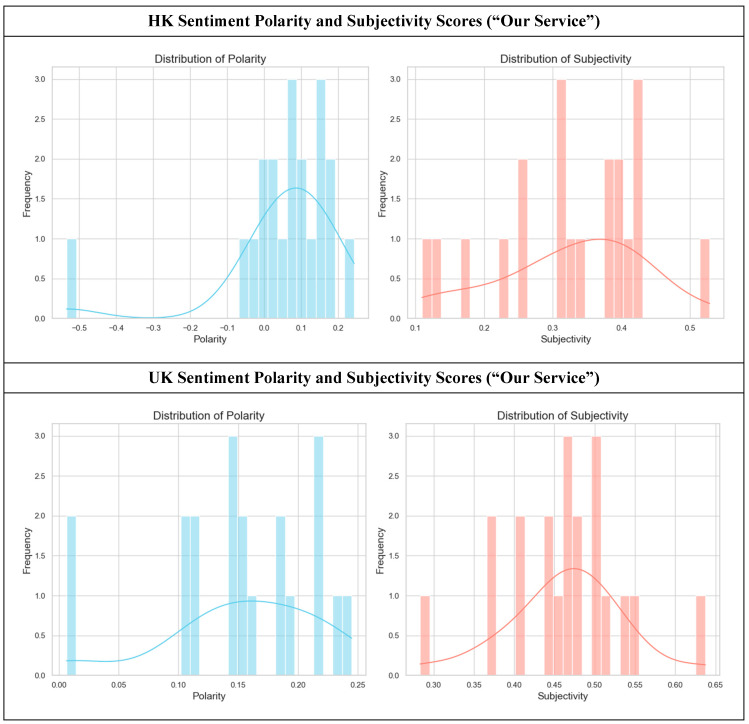
Sentiment polarity and subjectivity scores in hospice websites (HK “Our Service”, *n* = 20, vs. UK “Our Service” sections, *n* = 20). A Mann-Whitney U test showed that the UK “Our Service” pages had significantly higher sentiment polarity (*U* = 95.0, *z =* −2.84, *p =* 0.004703, *r =* 0.45). An independent samples *t*-test indicated that the UK had significantly higher subjectivity (*t*(38) = −4.72, *p* = 0.00003, Cohen’s d = 1.49) compared to the HK pages.

In the “Our Service” section, the language used to describe services also reveals differences in how hospice care is framed: as a solution to problems versus an enhancement of experience (Figure 4).

The HK “Our Service” website sections (top row, *n* = 20) adopt a practical, problem-solving tone. The polarity peaks at a modest positive value (M = 0.053, SD = 0.16) but notably includes some negative polarity scores. This suggests that the language focuses on addressing the negative aspects of illness, such as “pain management” or “alleviating discomfort”. The subjectivity is moderate (M = 0.33, SD = 0.11), indicating that services are described in a largely functional manner by emphasizing what they deliver rather than how they feel. The primary message is one of competent and practical medical support. In contrast, the UK corpus (bottom row, *n* = 20) frames services in an overwhelmingly positive and experiential light. The polarity is higher and more consistently positive (M = 0.15, SD = 0.06), while the subjectivity scores are elevated (M = 0.46, SD = 0.07).

A Mann-Whitney U Test confirmed that the sentiment polarity in the UK corpus was significantly higher than in the HK corpus (*U* = 95.0, *z* = −2.84, *p* = 0.004703, *r* = 0.45). This difference in subjectivity was also statistically significant (independent samples *t*-test: *t*(38) = −4.72, *p* = 0.00003, Cohen’s d = 1.49). This shows that UK websites likely move beyond function to describe the emotional and personal benefits of their services, using value-laden language such as “creating precious memories”, “enhancing well-being”, or “providing holistic comfort”. In summary, the HK approach in the ‘Our Service’ section is to present services as a professional solution to physical and logistical challenges. The UK approach is to portray services as a means to achieve emotional, personal, and holistic well-being, thereby focusing on the quality of life together with the management of illness.

Overall, the comparative sentiment analysis of hospice websites in HK and the UK reveals two divergent communication strategies for end-of-life care. The HK corpus consistently adopts a formal, objective, and reassuring tone. Across all sections, from institutional identity (“About Us”) to personnel (“Our People”) and offerings (“Our Service”), the language is characterized by low subjectivity and restrained positive polarity. This strategy builds trust by emphasizing professional competence, factual information, and practical, problem-solving support. In contrast, the UK corpus employs a relatively subjective, emotive, and personally engaging strategy. The language is more intensely positive and focuses on the character of its people and the experiential benefits of its services. By prioritising personal narratives and compassionate values, this approach aims to build trust by forging an immediate emotional connection and framing hospice care as a positive, life-affirming journey.

### 4.2. Conjunctions as Discourse Organizers Used in Hospice Websites

This section addresses the second research question, examining the role of conjunctions as key discourse organisers in hospice care websites in HK and the UK. Conjunctions are pivotal cohesive devices that signal logical, temporal, and semantic relationships between ideas, thereby helping to structure discourse effectively [37]. In the hospice context, conjunctions organise information and reflect communicative priorities and cultural values for a diverse audience. In this context, conjunction analysis involves a quantitative comparison and functional annotation of the discourse structure and culturally influenced styles of conjunctions used in digital health communication. Although the data are limited to publicly available website texts and do not include texts from individual families or patients, hospice practitioners can still benefit from learning how different patterns of conjunctions frame institutional care in an end-of-life context. The following two sections demonstrate how these conjunctions contribute to coherent discourse on digital hospice care.

#### 4.2.1. Addition “And” and Temporal “Once” Conjunctions in HK Hospice Websites

The high frequency and likelihood ratio of the addition conjunction “and” (5.48, *p* < 0.05) suggest that it fosters the connection of care and promotes the inclusivity of participants within the discourse. For example, the phrase “and families” frequently appears throughout the HK corpus, as shown in examples 1 and 2. This emphasizes the interdependence of patients and their families as care recipients, identifying this as a collaborative care framework.

(1) “The Centre provides rehabilitation programmes, self-care and life skills training to service users, as well as support for parents and families, ensuring they receive proper care and support.”
(2) “They provide community support for palliative patients and their families, complementing hospital services…”

From the perspective of service providers, patient support is not isolated, but part of an integrated continuum of care. Therefore, the conjunction “and” connects individual service provisions with whole-family support. This illustrates that effective patient care in HK is considered as encompassing familial involvement. This discourse strategy demonstrates the commitment of hospital services to well-being by inviting families to participate in the caregiving process. In addition, the repeated use of the conjunction “and” alongside the semantic pairing “patients and families” reinforces the idea that healthcare is a shared ethical responsibility, as demonstrated by example (3):

(3) “We fully understand the scale of emotional stress that our patients and their families face…”

This statement highlights the shared burden of care, reflecting the notion that emotional struggles during illness are a whole-family experience. It also emphasises the importance of family involvement in the caregiving process, including sharing both the care framework and the emotional burden.

Temporal conjunctions, particularly the term “once” (6.73, *p* < 0.01), highlight the procedural predictability emphasised on the HK hospice websites. The use of “once” emphasises the importance of reliable medical routines, reflecting a structured approach to care provision that aligns with societal values regarding order and predictability in hospice care, as illustrated in example 4.

(4) “The resident doctor conducts rounds once a week to carry out necessary treatment and control symptoms.”

By maintaining regular check-ups and predictable routines, this phrasing fosters a sense of trust and security among patients and families, thereby reinforcing the consistent nature of their service care.

#### 4.2.2. “Like” and “If” in UK Hospice Websites

UK hospice websites offer a different rhetorical strategy that diverges from the additive and procedural logic typically observed in HK hospice discourse, as discussed in Section 4.2.1. The conjunctions “like” (LL: 36.3) and “if” (LL: 33) are comparative and conditional conjunctions that build interpersonal meanings.

(5) “Personal care includes things like helping a person eat meals, wash, and take the time to shave or put on perfume…”

In example 5, “like” is used extensively in UK hospice texts to list daily activities in service centres, providing a comprehensive understanding of daily routines and helping to ease patients’ worries. Additionally, “shave” and “put on perfume” are considered respectful gestures that foster a sense of personal identity; thus, these phrases can evoke perceived intimacy, dignity, and care in readers. Another example found in the data reflects the provision of formal legal support, as shown in example 6:

(6) “They can help with things like organising a will, childcare, and a person’s last wishes.”

This broadens the scope of hospice care, shifting the focus from purely medical services to a more comprehensive approach that addresses both personal emotional needs and practical issues. Thus, these findings suggest that UK hospice service providers are patient-centred, emphasising empathy and tailoring their services to be flexible and responsive to individual needs [45].

“If” is a conditional conjunction that encourages dialogue and respects patients’ choices in decision-making.

(7) “If you would like to know more, please get in touch…”
(8) “If someone would like to stay with a patient overnight, we have extra beds…”

Example 7 is a typical example of low-pressure, respectful language, which is also a core principle of medical ethics, as it provides patients with a high degree of autonomy [46,47]. Example 8 emphasises the importance of anticipating patients’ emotional needs as part of compassionate care. We often found that “like” and “if” were paired with second-person pronouns such as “you” and “your”, as well as some emotionally resonant nouns such as “wants” and “wishes”. This communication strategy creates an empathetic tone, making the reader of the website an active participant in their hospice care journey rather than a passive recipient of care instructions. This strengthens the interpersonal dimension, fostering a sense of interconnectedness and approachability. Furthermore, phrases such as “like you might want” or “if you wish” make institutional boundaries more informal and encourage personal engagement in providing care tailored to the patient. This is also considered to be co-constructed care between the institution and patients [48]. In short, the approach to conjunctions is diverse across the two regions, with HK websites exhibiting a pronounced prevalence of additive and temporal conjunctions, fostering a very organized tone, and in contrast, UK websites use comparative and conditional conjunctions to create a more dialogic and personal, caring presentation in hospice communication.

Previous SFL studies on causal and additive conjunctions frequently examine their use in institutional texts, where they enhance informational and procedural clarity and organisational coherence see [34,35,37]. The patterns investigated in this study concerning the use of conjunctions across the two sub-corpora align with, and extend, these research studies on discourse structure to institutional and health communication. Building on this approach, the use of additive and temporal conjunctions on HK hospice websites illustrates a more structured and predictable healthcare service, a pattern that has been reported in earlier analyses of institutional healthcare discourse in Asian contexts see [49,50]. On the other hand, the predominant use of comparative and conditional conjunctions in the UK corpus is consistent with previous studies that have identified a more patient-centred and interactive discourse style in Western healthcare communication. In this style, institutional authority is realised through invitations, imaginative conditions, and negotiated language choices [34,48]. Therefore, findings from both regions suggest that conjunctions establish structural cohesion and reflect culturally related forms of engagement and positioning in end-of-life care.

### 4.3. Personal Pronouns and Interpersonal Meaning

Personal pronouns help to construct interpersonal meaning in hospice discourse by signalling the relationship between the producers and their receivers, establishing institutional identity, and influencing the style and inclusivity of communication [51,52]. In hospice care contexts, where emotional sensitivity and relational clarity are paramount, pronoun use plays an interactive role in developing and mediating relationships between care providers, patients, and families. This section examines the distribution and discursive function of personal pronouns on hospice websites in Hong Kong and the UK.

Table 2 shows the overall and relative frequencies, as well as the log-likelihood ratio, of the main personal pronouns in the two corpora. All differences discussed in the Findings and Discussion sections are statistically significant, as determined by log-likelihood testing with the thresholds specified in the previous Methodology section.

The UK hospice texts show significantly higher use of all measured pronouns, with particularly large disparities in the use of “our”, “you”, “she”, “I”, “we”, and “us”. All the log-likelihood values are statistically significant at *p* < 0.0001, reflecting the robust differences in interpersonal positioning between the two regions. The following examples suggest that HK texts adopt a more formal, organized, and service-oriented digital presentation style. Conversely, UK hospice texts encourage more personal storytelling and directly address the reader with an inclusive voice that transcends institutional boundaries.

Previous studies have shown that the first-person plural pronoun “we” is often used in institutional contexts to project organisational responsibility and institutional authority, whereas the second- and first-person singular pronouns tend to foreground narrative tone, personal involvement and patient-oriented alignment [41,53]. Thus, our findings regarding the use of personal pronouns are consistent with much research suggesting that pronouns are key indexical resources for constructing instructional identity, attitudes, and relational alignment in professional healthcare discourse [38,51,52].

To start with the use of personal pronouns in the Hong Kong texts, the predominant use of the pronoun “we” is frequent alongside the verb “offer” (LL: 26.7) in the following examples:

(9) “We offer a highly integrated service…”
(10) “We offer residential respite services…”

These pronoun strategies emphasise institutional professionalism and collaboration, presenting the hospice as a competent organisation. This is quite consistent with some Asian cultural norms, which prioritise respect for institutional capabilities over individual experiences and personal discourse in formal institutional communication [49,50]. The collocation “we strive” signals a transition from professional service provision to aspirational discourse, highlighting the hospice’s vision and moral commitments.

(11) “We strive to help our patients find comfort.”

This example demonstrates the institution’s ethical commitment to higher principles, not only in improving clinical outcomes but also in addressing the emotional and spiritual needs of patients. Furthermore, the phrase “we + encourage” (LL: 16.6) inspires active participation from patients and their families.

(12) We also encourage families to conduct life reviews with elderly relatives, recognising their previous efforts and contributions.

Such activities facilitate emotional healing and support shared decision-making, as observed in contexts that promote advanced care planning. From the three significant collocational pronoun pairs above, we recognise the triadic institutional voice comprising capabilities, principled values, and participatory engagement. This enables HK hospices to balance authority with personal agency while demonstrating cultural sensitivity and understanding.

The personal pronoun “our” is frequently used in the UK hospice websites, and the most dominant and significant collocations include “our + team”, “our + inpatient”, “our + community”, and “our + patients”. In particular, “our team” (LL: 102.4) fosters a sense of personification, creating interpersonal connections between the writers and readers.

(13) “Our team includes family support workers, therapists, and counsellors…”
(14) “We encourage you to treat our Inpatient Unit as you would your own home.”

Example 13 shows the multidisciplinary care models employed by the service centres, which provide collaborative support to patients. The collocation “our inpatient” (LL: 86.4) also frames the institutional space as familiar and comfortable, as shown in Example 14. This language explicitly merges the boundaries between clinical and domestic spaces, promoting a sense of belonging and patient autonomy. The collocations “our community” (LL: 55.9) and “our patients” (LL: 37.4) in the UK hospice websites create an interpersonal voice for the institutions. Specifically, “our community” embeds hospice care into local life, highlighting community and group solidarity. Example 15 shows an appreciation for the support from the community that contributes to the hospice care.

(15) “Our services are free… made possible through the generosity of our amazing community.”
(16) “Wearing PPE masks has been difficult for some of our patients…”

Example 16 uses “our patients” to describe the shared experience of the arrangement between carer and patient. These pronoun patterns reflect an inclusive approach towards patients [53,54]. Thus, the possessive pronoun “our” signals ownership and inclusion, which aligns with British cultural values of modesty and empathy [55]. This contrasts slightly with the pattern in the Hong Kong dataset, where the “we + offer” formulation is more formal and emphasises institutional responsibility and professionalism. However, it should be noted that these pronoun patterns cannot be interpreted as evidence of the lived experience of individual practitioners or patients, as they do not represent direct interaction. They should therefore be understood as representations of organisational attitude.

The use of phrases such as “you + medicines”, “you + welcome”, and “you + judging” on UK hospice websites indicates an approach to language that respects patient autonomy and expresses emotional support. Regarding medicines, patients are presented as informed participants.

(17) “Please bring all tablets and medicines you are currently taking.”
(18) “We will make sure you have all the medicines you need…”

These language patterns deliver polite and gentle reminders to patients and prioritise clear listing, demonstrating a high level of transparency and continuity of care. Additionally, the phrase “you’re welcome” fosters a permissive environment within centres.

(19) “You’re welcome to bring personal items from home…”

Similarly, example 20 reads as follows:

(20) “Your visitors are welcome to eat with you.”

Such language patterns permit and encourage patients to bring personal belongings into clinical settings, which can transform these public spaces into safer and much more private environments. In example 20, the writer incorporates family daily life (“eating with you”) into the hospice care experience, which demonstrates a high level of human warmth and interpersonal support that goes beyond institutional settings. Finally, we found an interesting example, which is rare but powerful, shown in example 21: “without judging you”. This signals a kind of unconditional acceptance and understanding of the patient from the service provider in this therapeutic context.

(21) “We accept the way you are, without judging you.”

This establishes the hospice as a safe emotional space, with staff providing great care to their patients, contrasting with the usual clinical hierarchy in which clinical staff have a higher status. We consider this to be emotionally attuned care that is deeply humanising and necessary for hospice patients.

The personal pronoun “I” appears frequently alongside the phrases “I was impressed” and “I knew” on the UK hospice websites. These phrases are used by patients to share their experiences, expressing gratitude and appreciation for the centre.

(22) “I have been consistently impressed by the standard of care…”
(23) “I knew this was the place for me because they served steak bakes…”

“I was impressed” is an emotional endorsement from patients, showing appreciation for the hospice’s care practices and supporting its credibility and goodwill, as shown in example 22. Such expressions demonstrate a different perspective that can foster authenticity and trust. In addition, “I knew” often marks a turning point in personal narratives, allowing a more relaxed and personal tone to emerge naturally. In example 23, choosing the right hospice can be as simple as being attracted to steak bakes, which help to ease patients’ worries and fears. This pronoun strategy is a care model constructed within a communicative strategy.

“We + offer” patterns in the Hong Kong corpus align with findings from healthcare communication in institutional contexts that focus on service quality, competence, and professionalism [49,50]. Our findings are also consistent with previous studies that have illustrated the significance of empathy and interpersonal closeness in hospice communication [1,2]. In contrast, the frequent use of “you”, “our” and “I” in institutional discourse personalises it, blurring the boundaries between the patient experience and the organisational voice. These subtle grammatical language choices shape expectations of care, alignment, and trust in end-of-life contexts. Thus, the present study contributes to this field by identifying the importance of empathy and how distinct pronoun resources operate as interpersonal strategies suited to digital health communication.

Recent socio-technical research in digital health has highlighted that digital communication tools are largely shaped by broader social and institutional contexts [56]. However, the effective dissemination of information is often affected by different practices and institutional assumptions [57,58]. In particular, minority populations, such as migrants and refugees, have faced challenges related to language barriers and differing levels of digital and health literacy. This has resulted in uneven access to devices, and unequal access to internet connectivity and usage [56,59]. While this study does not directly address migrant-specific hospice services, from this perspective, hospice websites are understood as socio-institutional texts whose language choices, which are constructed under certain assumptions about their target audience, how information is framed, and how users are expected to engage with it. This socio-technical approach is thus consistent with the present findings, which illustrate how patterns of interpersonal meaning can systematically position readers in culturally specific ways across different datasets.

In summary, we have discussed how sentiment, conjunctions, and personal pronouns contribute to hospice communication in Hong Kong and the UK. Our data show that Hong Kong websites adopt a more formal and objective tone, using less emotive language and sequential conjunctions to emphasise professionalism and structured care services. In contrast, UK websites tend to use more emotive language and inclusive pronouns, such as “you”, “our”, and “I”, to foster empathy, personal connection, and patient autonomy.

## 5. Conclusions

The language used in hospices is deeply rooted in professional practices and cultural communication norms [1,60]. At a macro level, sentiment analysis shows that Hong Kong hospice websites tend to focus on procedural clarity, factual competence, and institutional authority to construct an institutional identity, while UK hospice websites focus more on relational warmth and shared values to foster immediate emotional connections. These various orientations are also reflected at a micro level through lexicogrammatical features: for example, HK texts use more additive and causal conjunctions, while UK texts use more concessive and conditional conjunctions and inclusive pronouns. These findings demonstrate that sentiment, conjunctions, and personal pronouns are interconnected lexicogrammatical resources through which hospices digitally construct “good death” support.

Theoretically, this NLP-based sentiment analysis with Systemic Functional Linguistics takes a systematic mixed-methods approach to investigating culturally shaped digital health communication. It illustrates how computational techniques can complement qualitative analysis in revealing interpersonal meaning in institutional healthcare discourse. In practice, this study examines hospice websites that actively shape the experience of interpersonal alignment between professionalism and empathy in culturally situated texts. This has direct implications for how hospice content is framed and tailored to help patients, families, and caregivers navigate the end-of-life journey. Lastly, in terms of limitations, this study only focused on English hospice websites in two regions and did not address multilingual communication practices or the interpretations of individual users. Building on this approach, future research could explore more qualitative methods, such as investigating how patients, families, and carers engage in digital hospice communication in different contexts.

## Figures and Tables

**Table 1:** External conjunctions [34] (p. 153).

Types			Examples
addition	additive	adding	and, besides, both… and
		subtracting	nor, neither… nor
	alternative		or, either… or, if not… then
comparison	similar		like, as if
	different	opposite	whereas, while
		replacing	instead of, in place of, rather than
		expecting	except that, other than, apart from
time	successive	sometime	after, since, now that, before
		immediate	once, as soon as, until
	simultaneous		as, while, when
cause		expectant	because, so, therefore
		concessive	although, even though, but, however
means		expectant	by, thus
		concessive	even by, but
condition	open	expectant	if, then, provided that, as long as
		concessive	even if, even then
	closed		unless
purpose	desire	expectant	so that, in order to, in case
		concessive	even so, without
	fear		lest, for fear of

**Table 2:** Frequency and log-likelihood values of personal pronouns in Hong Kong and the UK hospice websites.

Words	Raw Freq. in HK Websites	Relative Freq. in HK Websites	Raw Freq. in UK Websites	Relative Freq. in UK Websites	Log- Likelihood Ratio (LL)	*p*-Value
1. our	81	61	618	159	83.20	*p* < 0.0001
2. you	30	23	312	80	61.99	*p* < 0.0001
3. I	3	2	104	27	41.91	*p* < 0.0001
4. we	85	64	485	125	37.29	*p* < 0.0001

## Data Availability

The data for this study consists of official hospice websites that are publicly available online. While the source websites are publicly accessible, the specific corpus compiled, processed, and analyzed for this study constitutes a curated research dataset. To protect the integrity of the study and the context of the data, this dataset is not placed on an open-access platform. Requests for further details regarding the data will be considered by the corresponding author, subject to a formal ethical review.
